# Development of Optical Differential Sensing Based on Nanomaterials for Biological Analysis

**DOI:** 10.3390/bios14040170

**Published:** 2024-03-31

**Authors:** Lele Wang, Yanli Wen, Lanying Li, Xue Yang, Wen Li, Meixia Cao, Qing Tao, Xiaoguang Sun, Gang Liu

**Affiliations:** Key Laboratory of Bioanalysis and Metrology for State Market Regulation, Shanghai Institute of Measurement and Testing Technology, 1500 Zhang Heng Road, Shanghai 201203, China; wangll@simt.com.cn (L.W.); lily@simt.com.cn (L.L.); yangxue@simt.com.cn (X.Y.); liw@simt.com.cn (W.L.); caomx@simt.com.cn (M.C.); taoq@simt.com.cn (Q.T.); sunxg@simt.com.cn (X.S.)

**Keywords:** pattern recognition, nanomaterials, gold nanoparticle, graphene oxide, quantum dot

## Abstract

The discrimination and recognition of biological targets, such as proteins, cells, and bacteria, are of utmost importance in various fields of biological research and production. These include areas like biological medicine, clinical diagnosis, and microbiology analysis. In order to efficiently and cost-effectively identify a specific target from a wide range of possibilities, researchers have developed a technique called differential sensing. Unlike traditional “lock-and-key” sensors that rely on specific interactions between receptors and analytes, differential sensing makes use of cross-reactive receptors. These sensors offer less specificity but can cross-react with a wide range of analytes to produce a large amount of data. Many pattern recognition strategies have been developed and have shown promising results in identifying complex analytes. To create advanced sensor arrays for higher analysis efficiency and larger recognizing range, various nanomaterials have been utilized as sensing probes. These nanomaterials possess distinct molecular affinities, optical/electrical properties, and biological compatibility, and are conveniently functionalized. In this review, our focus is on recently reported optical sensor arrays that utilize nanomaterials to discriminate bioanalytes, including proteins, cells, and bacteria.

## 1. Introduction

In recent decades, natural/artificial specific receptors have been studied for the analysis of particular analytes based on the lock-and-key principle in many critical fields, including food safety [1,2,3,4,5,6,7,8,9,10,11], environmental monitoring [12,13,14,15,16,17], and medical diagnosis [18,19,20,21,22,23,24,25,26,27,28,29,30,31]. However, the production of highly specific receptors remains a challenge for a large range of analysis targets, especially when facing complex biological samples containing proteins, microorganisms, and cells.

**Scheme 1:**
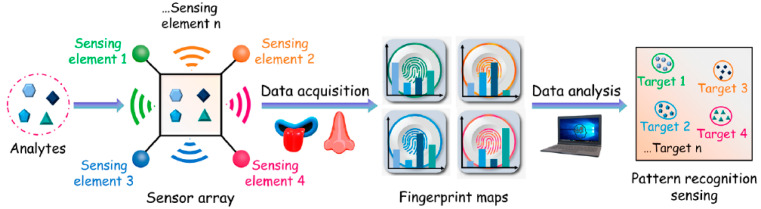
Mechanisms of Sensor Arrays for Discrimination and Identification of Analytes. Reprinted with permission from [33]. Copyright 2023 American Chemical Society.

Recently, pattern recognition has been intensively studied, also known as differential sensing or “artificial noses/tongues” [32,33,34,35,36,37]. Different from traditional molecular recognition based on one specific receptor, differential sensing was constructed on a receptor library of low-specific recognizing elements, each of which would respond to a certain target to different degrees [38,39,40]. By collecting the response signals, we can establish a fingerprint toward characteristic patterns for the individual analytes or complex mixtures. To perform differential sensing, a sensor array was constructed as the central component. Through array analysis, data from various sensing units could be gathered concurrently and subsequently scrutinized to facilitate target detection and recognition (Scheme 1). The number of channels within the array is a crucial factor influencing the discrimination capacity of the differential sensor. An illustrious example highlighting this principle is the olfactory system of a dog, which possesses approximately 4 billion olfactory receptor cells, an astonishing 45 times more than that of a human. The signals detected by these receptors have the potential to generate even larger quantities of interconnected data groups through their intricate associations with one another.

There are two main obstacles to the development of artificial sensors: Firstly, it is difficult to construct a large-scale array to collect adequate signals compared with natural systems. Secondly, the sensitivity is usually hindered by the relatively high blank noise signal or low signal read-out, especially in biological samples. Thus, there have been increasing research demands to develop novel biosensing strategies for higher sensitivity and larger scale of sensor arrays [37]. In recent decades, nanomaterials have become a shining star in the research of a growing number of biosensor strategies [41,42,43,44,45,46,47,48,49,50]. The emergence of fast-growing nanomaterials [51], such as metal nanoparticles [48,52,53,54,55,56], carbon nanomaterials [44,46,57,58], and quantum dots [59,60,61], has opened up exciting possibilities for novel sensor platforms [62,63,64]. These nanomaterials possess unique electronic, magnetic, and light properties, making them highly desirable for the field of differential sensing. Table 1 displays the main characteristics of the common nanomaterials studied for optical differential sensing.

In this review, we present an overview of the applications of functional nanomaterials in optical sensor arrays, including colorimetric and fluorescence methods. These arrays can be categorized into gold nanoparticle-based sensor arrays, graphene oxide (GO)-based sensor arrays, quantum dot (QD)-based sensor arrays and other metal nanoparticle-based sensor arrays. Table 2 presents the timeline for the historical development of optical differential sensing based on nanomaterials for biological analysis. Compared to the former literature, this review aims to provide a comprehensive understanding of the advancements, challenges, and future prospects in this rapidly evolving field. We here mainly focus on three main significant advantages and contributions of nanomaterials for the development of sensor arrays: Firstly, by manipulating their physical and chemical properties as well as surface modifications, functional nanomaterials enhance signal output, sensitivity, and selectivity. Secondly, the unique properties and interaction mechanisms of functional nanomaterials enable sensor arrays to detect multiple target molecules and achieve multiparameter analysis. Additionally, functional nanomaterials allow for efficient analysis of complex samples by integrating multiple sensing mechanisms such as fluorescence resonance energy transfer and surface plasmon resonance. Thus, the integration of functional nanomaterials into sensor arrays holds great promise in advancing the field of optical sensing, offering new avenues for exploring various detection technologies and expanding the range of potential applications. 

## 2. Pattern Recognition Methods for Differential Sensing

**Scheme 2:**
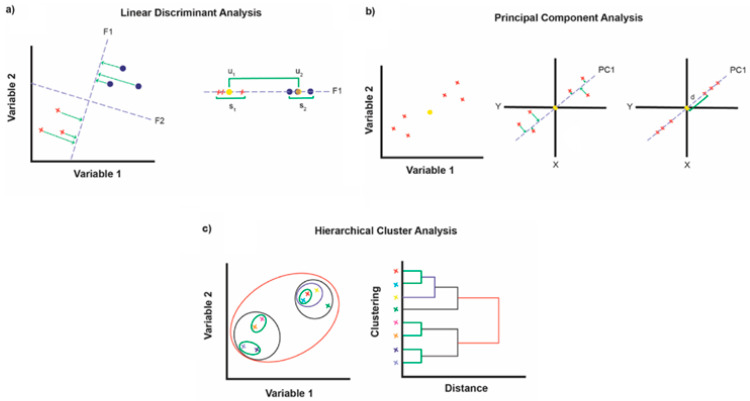
Schematic representation of (**a**) the LDA method of projecting points onto a new vector F1 that fulfils the criteria of maximizing the ratio of between-class to within-class variance, (**b**) the PCA method of determining the center of the data, projecting points onto a new vector, and calculating the maximum variance and thus the best-fitting line, (**c**) the HCA bottom-up agglomerative approach and the resulting dendrogram illustrating the connectivity of data points. Reprinted with permission from [85]. Copyright 2021 American Chemical Society.

Optical signals produced by the differential sensing array were analyzed by using pattern recognition methods such as linear discriminant analysis (LDA) [79], principal component analysis (PCA) [80], and hierarchical clustering analysis (HCA) [81]. A schematic representation of the above methods is shown in Scheme 2.

Linear discriminant analysis is a supervised pattern recognition method that can be used for both dimensionality reduction and classification [82]. The means and covariance matrices of the training data set are used to establish the discriminant functions. Once the discriminant functions are built, a prediction data set is tested by the discriminant functions to validate the classification accuracy. In order to ensure classification accuracy, the prediction data set should be different from the training data set; otherwise, LDA may produce optimistic results.

Principal component analysis (PCA) is an unsupervised method for dimensionality reduction of multivariate data. It can compress a multi-dimensional data set into a lower dimensional space and rank the new dimensions according to their importance. Often, a successful PCA may produce two or three principal components, which are convenient for producing score plots for the data set [83]. It is important to note that PCA is more suitable for the analysis of linear data; however, it is possible to fail the classification of nonlinear data.

Similar to PCA, hierarchical clustering analysis (HCA) is an unsupervised pattern recognition method. There are three basic steps for HCA: Firstly, the multivariate distances between all samples are calculated. Afterward, clustering is performed by establishing a hierarchy of points, in which similar distant points are joined. Finally, a two-dimensional dendrogram is shown that allows the visual examination of clustering relationships of all samples [84]. Because HCA employs all the sensor array data to represent the patterns, a poor result may be produced when the data set is noisy. HCA is most suitable for qualitative analysis of relationships in data.

## 3. Gold Nanoparticle-Based Sensor Arrays

Gold nanoparticles (AuNPs) have been widely studied in the development of biosensors due to their unique optical and chemical properties, good biocompatibility, and easy surface functionalization [86,87,88]. Together with organic or biological molecules, AuNPs can produce differential response signals for target molecules [65,67,74,89,90,91,92,93,94,95,96,97,98,99,100,101,102,103,104,105,106,107,108,109,110,111,112,113].

### 3.1. Fluorescence Sensing Based on AuNPs

AuNPs are widely applied in biosensors as powerful fluorescence quenchers [114,115,116,117,118,119,120]. The competitive bindings between the analytes and the indicators to AuNPs lead to distinct fluorescence response fingerprints for many analytes, which could be identified by pattern recognition methods with a high degree of accuracy [121]. These AuNPs work as powerful fluorescence quenchers for fluorescence indicators, as well as the recognition elements for target analytes. The interactions between nanoparticle–indicators and nanoparticle-analytes could be tuned by modifying different groups on the surface of AuNPs.

A sensor array was developed for the differentiation of normal and cancerous cell lines, based on conjugates between three structurally related cationic AuNPs and the fluorescent polymer [90,122]. The nanoparticles quench the fluorescence of the polymer. In the presence of mammalian cells, there is competitive binding between nanoparticle-polymer complexes and cell types. The polymer was displaced with mammalian cells from the nanoparticle surface, generating a fluorescence response. Four different types of human cancer cells were discriminated by using LDA. The results showed a 100% accuracy of detection. The sensor array can also effectively differentiate isogenic cell types. Later, the same group designed a sensor array composed of AuNP-GFP complexes for discrimination between normal and metastatic cells and tissues [67]. Rather than using whole cells as the target analytes, the lysates isolated from tissues have the advantage of increased homogeneity of the test samples, which leads to reduced error in identification, increased reproducibility, and higher sensitivity. This sensing platform needed a small amount of sample (as little as 200 ng of cell- or tissue-lysed proteins).

**Figure 1:**
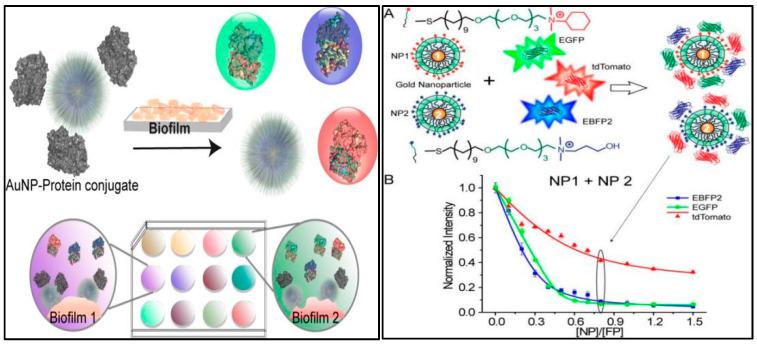
(**Left**) Schematic illustration of the multichannel sensor using AuNP-fluorescent protein conjugates that are disrupted in the presence of biofilms. (**Right**) The sensor composition. (**A**) Sensor elements and molecular structures of the functional ligands of NP1 and NP2. (**B**) Fluorescence titration with an equal molar mixture of NP1 and NP2. Reprinted from [70] with open access.

The Rotello group synthesized two types of AuNPs, one with a cationic hydrophobic functional group and the other with a hydrophilic functional group [70] (Figure 1). Three fluorescent proteins with negative surface charge can bind to these particles through electrostatic interactions, resulting in fluorescence quenching. When exposed to bacteria biofilms, AuNP-fluorescent protein conjugates are disrupted to produce different colored fluorescence patterns. The multichannel sensor was able to completely differentiate six bacterial biofilms, including nonpathogenic and pathogenic bacteria. The performance of the sensor was further tested by the identification of biofilms in a mixed bacteria/mammalian cell in vitro wound model.

### 3.2. Colorimetric Sensing Based on AuNPs

The aggregation of AuNPs results in a visible color change from red to blue, which provides a versatile platform for colorimetric sensing of target analytes [96,123,124,125]. Zhang and co-workers created a colorimetric sensor array with aptamer-protected AuNPs as recognition elements [126]. The aptamer-protected AuNPs were able to resist aggregation in the presence of a high-concentration salt. Upon the addition of different target proteins, differential response patterns were obtained. This sensitive array sensing system can discriminate seven proteins with the naked eye at the 50 nM level. Similar approaches were also used for the analysis of many bioanalytes [127,128,129]. These sensor arrays exhibited an excellent ability to recognize proteins, bacteria, and mammalian cells.

Chen et al. constructed a DNA-AuNPs colorimetric sensor array for rapid and sensitive identification of proteins [128]. The sensor array composed of only two sensing elements could discriminate 12 proteins at the 50 nM level with the naked eye. Moreover, the proteins in human serum and protein mixtures were well-differentiated with 100% accuracy. Huang and co-workers also exploited DNA-AuNPs nanoconjugates to differentiate cell types [129]. The cross-reactive receptors (DNA-AuNPs) are employed to bind the different cells that produce differential color changes of AuNPs. The nanoplasmonic effect of AuNPs was enhanced via seeded growth, which resulted in the effective distinction of various cell lines with dark-field microscopy or even the naked eye. The results were analyzed by LDA, which showed 100% accuracy.

Wu and Shi [28] developed a colorimetric sensor array for rapid microorganism identification. The array utilized four distinct AuNPs as sensing elements, resulting in noticeable color shifts upon interaction with microorganisms. Through LDA, 15 microorganisms were successfully differentiated based on their unique response patterns. The sensor array also demonstrated the ability to discern mixtures of microorganisms. This straightforward and expedient method provides results within 5 s, making it suitable for applications in pathogen diagnosis and environmental monitoring.

**Figure 2:**
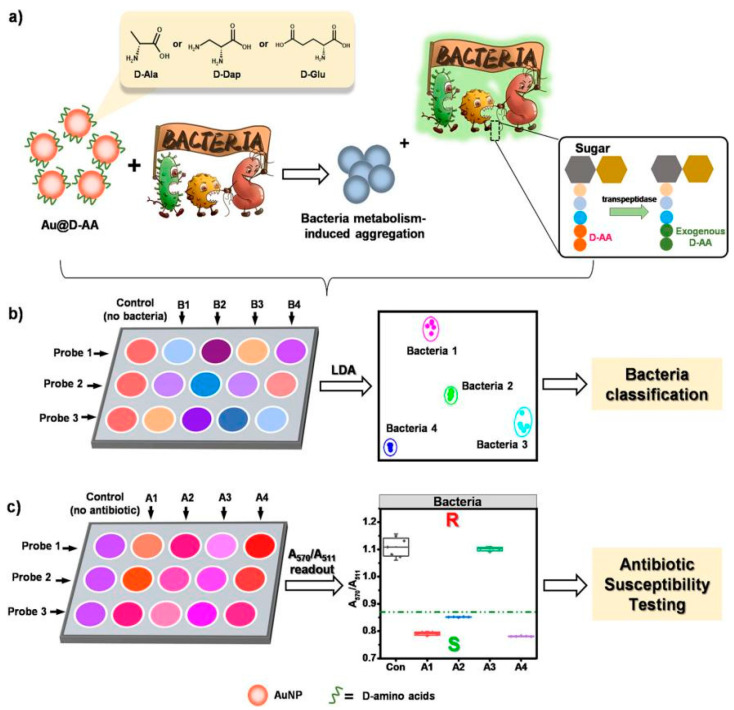
Principle of the Developed Assay Based on the Bacteria Metabolism-Triggered Consumption of dD-AA (**a**), Strategies for Multiple Bacteria Identifications through LDA (**b**) and AST through the Colorimetric Change of Probes after the Incubation with Bacteria and Antibiotic (**c**). Reprinted with permission from [130]. Copyright 2022 American Chemical Society.

A colorimetric sensor array was developed using D-amino acid (D-AA)-modified AuNPs as probes (Au/D-AA) for bacteria fingerprinting [130]. The aggregation of AuNPs is triggered by the metabolic activity of bacteria towards D-AA, allowing differentiation of eight types of bacteria and quantitative analysis of a single bacterium. The sensor array also enables rapid colorimetric antibiotic susceptibility testing (AST) by monitoring bacterial metabolic activity toward different antibiotic treatments, which has implications for clinical applications and antibiotic stewardship (Figure 2).

Liu and co-workers presented an extensible multidimensional sensor using the conjugates of nonspecific dye-labeled DNA sequences and AuNPs as receptors [127]. The changes in the fluorescent and colorimetric signals were generated by the addition of the target proteins due to the competitive binding. The array has a strong ability to distinguish 11 protein analytes with a detection limit as low as 50 nM. Also, 10 proteins at 1.0 μM were well-identified when the proteins were spiked into the human urine sample.

### 3.3. Differential Sensing Based on Gold Nanoclusters (AuNCs)

More recently, AuNCs have attracted much interest in biosensing applications [131,132,133,134]. Compared with semiconductor quantum dots or other metal NDs, AuNCs possess several distinct features, such as photophysical/chemical properties, good stability, and excellent biocompatibility [135,136,137,138,139,140,141,142]. Several studies utilized AuNCs for the construction of differential sensing strategies [72,143,144,145]. Ouyang and co-workers designed a visual sensor array based on blue-emitting Col-AuNCs and Mac-AuNCs for the discrimination of proteins [70]. The colorimetric and fluorometric signal changes were recorded after the addition of the target proteins. Either or both proteins and protein mixtures after polyacrylamide electrophoresis were well-discriminated by LDA. 

Luo’s group also developed a protein sensing platform using six dual ligand functionalized AuNCs as sensing receptors [144], by functionalizing them with different amino acids. When they compared the relative fluorescence changes with the LDA method, ten proteins were successfully discriminated. Wu and co-workers [146] developed a fluorescence sensor array based on metal ion-AuNCs for the identification of proteins and bacteria. The sensor array successfully differentiated nine proteins with different concentrations and identified five different types of bacteria, demonstrating its potential for rapid and sensitive biomolecule sensing.

**Figure 3:**
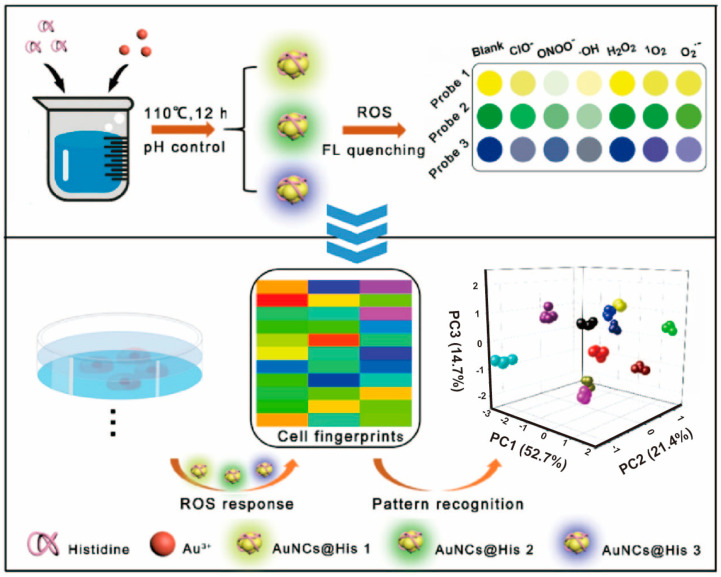
Schematic diagram of precise diagnosis of cancer via an ROS-responsive fluorescent sensor array based on pH-controlled multicolor histidine-templated AuNCs. Reprinted with permission from [147]. Copyright 2023 American Chemical Society.

A pH-controlled histidine-templated AuNC (AuNCs@His) [147] was developed for a fluorescent sensor array that responds to reactive oxygen species (ROS) for distinguishing cancer cell types and their proliferation states. The sensor array exhibited excellent performance in accurately differentiating cancer cell types and their proliferation states, indicating great potential for precise cancer diagnosis (Figure 3). Li and Zhu [148] developed a multichannel sensor array for efficient identification of bacteria based on three antimicrobial agents (vancomycin, lysozyme, and bacitracin) functional AuNCs. This sensing platform successfully differentiated seven pathogenic bacteria, different concentrations of the same bacteria, and even bacterial mixtures, offering a rapid and reliable method for diagnosing urinary tract infections.

In summary, gold nanoparticles are good candidates for the development of sensor arrays for biological analysis, and the main characteristics of the different sensor arrays are shown in Table 3.

## 4. Graphene Oxide (GO)-Based Sensor Arrays

GO is a chemically exfoliated graphene derivative, which can be utilized as a fluorescence quencher for various fluorescent probes, such as fluorescent polymer [76,151,152], fluorescent protein [68], metal nanodots [153], and fluorescently labeled DNA [69,154,155,156,157,158]. More importantly, GO showed differential affinity toward different molecules or materials [159,160]. Thus, GO has been widely applied as an ideal artificial receptor for the construction of nose/tongue sensors [71,75,150,161,162,163,164,165,166], as shown in Table 4.

The differential sensor for protein detection was developed based on GO [68]. Initially, fluorescent reporters (eGFP, pyronin Y, rhodamine 6G, acridine orange, rhodamine B) were quenched when combined to GO, and then different proteins could displace the fluorophores and restored different levels of fluorescence signal according to the affinity between GO and the proteins. In their work, a novel kind of nanoscale GO (nGOs) with a near-uniform dimension of 20 nm was applied, showing much better recognition capability than conventional GO, because nGOs have a higher supramolecular response and replacement rate. Their results showed that the nGO arrays can discriminate eight different proteins at 100 nM and 10 nM, and the success rate was as high as 95% when analyzing 48 unknowns.

Fan and co-workers combined the adaptive “ensemble aptamers” (ENSaptamers) and nGOs to develop a sensor array for high-precision identification of proteins, bacteria, and cells [69]. Auguste and co-workers provided a sensing array for the identification of healthy, cancerous, and metastatic human breast cells using six luminescent nanodot-graphene oxide complexes as novel fluorescent nanoprobes [153]. The sensing system was disrupted in the presence of breast cells, producing a distinct fluorescence response pattern. The multichannel sensor was capable of effectively identifying healthy, cancerous, and metastatic human breast cells with as few as 200 cells. Tomita and co-workers constructed a cross-reactive DNA-based array for one-step identification of antibody degradation pathways. The signature-based sensing platform was able to identify a broad range of degraded antibodies, such as common features of native, denatured, and visibly aggregated antibodies, complicated degradation pathways of therapeutic omalizumab upon time-course heat-treatment, and the individual compositions of differently degraded omalizumab mixtures. Tang and Qin [167] developed a microbial lysate-responsive fluorescent sensor array using luminogens featuring aggregation-induced emission characteristics (AIEgens) and graphene oxide (GO). This combination effectively reduces background signals and enhances discrimination ability through competitive interactions among AIEgens, microbial lysates, and GO. The sensor array successfully identified six microbes, including fungi, Gram-positive bacteria, and Gram-negative bacteria.

**Figure 4:**
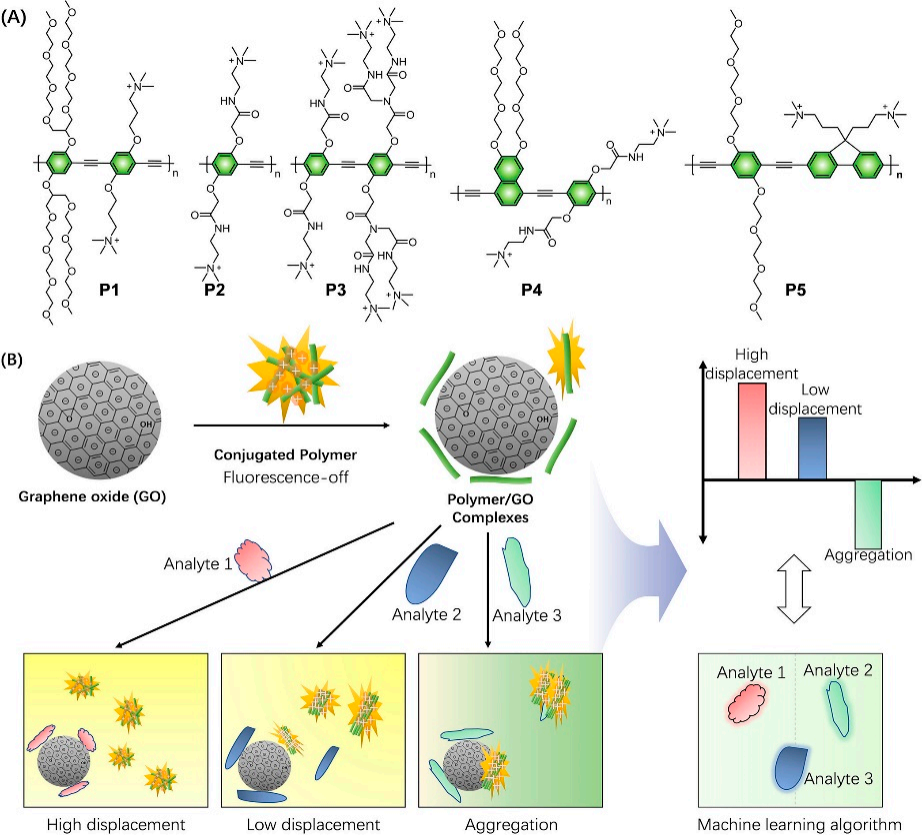
(**A**) Chemical structures of fluorescent polymers P1–P5. (**B**) Schematic illustration of the construction of electrostatic complexes from positively charged poly(para-aryleneethynylene)s and negatively charged GO and identification mechanism for multiple analytes. Reprinted with permission from [76]. Copyright 2022 American Chemical Society.

Han and co-workers [77] developed a novel multichannel array using modified polyethyleneimine and graphene oxide. This complex system enabled the successful identification of 10 bacteria within minutes through electrostatic and hydrophobic interactions. The sensor array also demonstrated the ability to measure bacterial concentrations and identify mixed bacteria accurately. In biological samples such as urine, the array achieved high accuracy. Han and co-workers [76] also designed five positively charged poly(para-aryleneethynylene) (P1–P5) molecules to form electrostatic complexes (C1–C5) with negatively charged graphene oxide (GO), effectively distinguishing between 12 proteins while employing machine learning algorithms. Moreover, these sensor arrays accurately identified levels of Aβ40 and Aβ42 aggregates, including monomers, oligomers, and fibrils, offering an attractive strategy for early Alzheimer’s disease diagnosis (Figure 4).

## 5. Quantum Dot (QD)-Based Sensor Arrays

**Figure 5:**
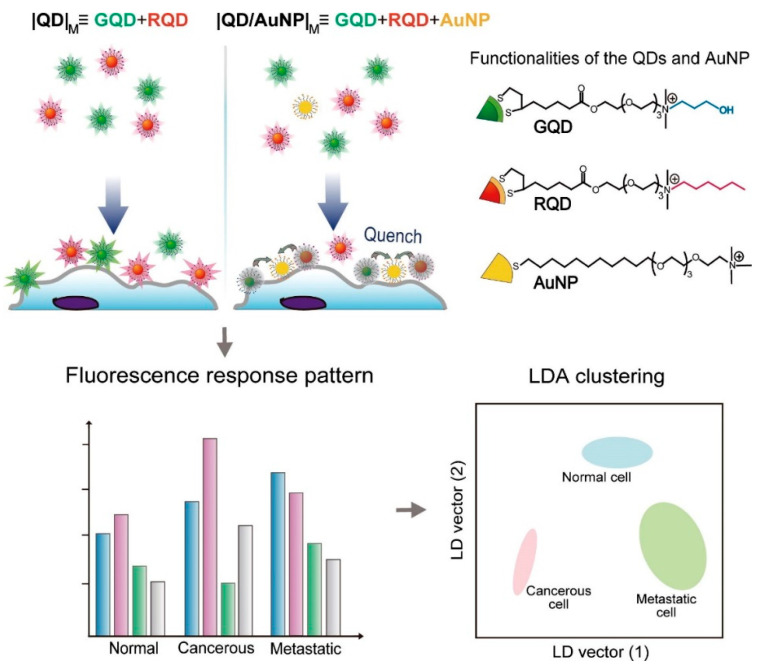
Illustration showing how nanoparticles interact with the cell surface in a sensing system, resulting in differential quenching and distinct patterns for distinguishing different cell types/states. Reprinted with permission from [100]. 2012 Elsevier Ireland Ltd. All rights reserved.

Based on their distinguished characteristics of good photostability, high quantum yield, and long fluorescence lifetime, QDs have been extensively used in fluorescent bioanalysis [161,168,169,170,171]. Rotello and co-workers developed a QD-based sensor for sensing mammalian cell types and states [100]. The sensing system is composed of two quantum dots and one gold nanoparticle. The quantum dots serve as transducers, which can be quenched by the gold nanoparticle. Different cell types and states were successfully differentiated by the sensor array (Figure 5).

Wang and Chen developed a fluorescent sensor array using imidazolium ionic liquids (ILs) and ionic liquid-QD conjugates as semi-selective receptors for the discrimination of proteins [172]. The IL sensing system was able to differentiate eight proteins at a concentration of 500 nM with an accuracy of 91.7%. With the improvements of the sensitivity and discrimination accuracy, the IL@QDs/QDs sensing system could distinguish eight proteins with 100% accuracy at a very low concentration of 10 nM. Additionally, protein mixtures and proteins spiked in human urine were well-discriminated by the IL@QDs/QDs sensing system.

Yan and co-workers designed a multidimensional sensing device based on Mn-ZnS QDs for the discrimination of proteins [173]. The triple-channel optical properties (fluorescence, phosphorescence, light scattering) of Mn-ZnS QDs were utilized to achieve the output signals. After interaction with target proteins, the changes in the triple-channel optical properties of Mn–ZnS QDs were observed. The multidimensional sensing devices were able to generate distinct patterns for different proteins. Eight proteins added to human urine samples were successfully discriminated against with the aid of principal component analysis.

**Figure 6:**
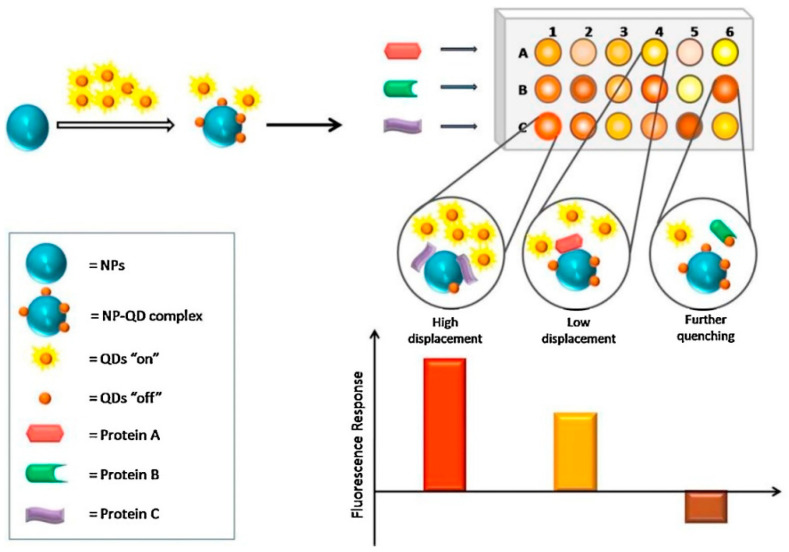
Schematic illustration of a fluorescence sensor array based on six types of NP-QD complexes. Reprinted with permission from [174]; 2017 Elsevier B.V. All rights reserved.

Combination of different nanomaterials, Wu and Zhang developed a nanoparticle quantum dot-based fluorescence sensor array for sensing proteins and cancer cells [174]. The sensor array consists of six types of nanoparticles (NPs, including CuO, ZnO, Eu_2_O_3_, AuNPs, AgNPs, Au-Ag core-shell) and CdSe quantum dots (Figure 6). These NPs can quench the fluorescence of CdSe quantum dots. The NP-QD interaction was disrupted by the addition of proteins, leading to fluorescence turn-on or further quenching. Eight proteins were readily differentiated by using LDA analysis. Moreover, protein quantification was achieved with the limits of detection below 2 μM in the range of 2–50 μM. Qu and Ren [71] designed seven fluorescent luminescent nanoprobes, including graphene quantum dots (GQDs), CdTe quantum dots (QDs), carboxyl-carbon dots (CDs-COOH), polyethyleneimine functionalized carbon dots (PEI-CDs), BSA-templated gold nanoclusters (BSA-AuNCs), lysozyme-templated gold nanoclusters (LysAuNCs), and DNA-templated silver nanoclusters (AgNCs), and they used graphene oxide (GO) as an excellent quencher with different affinity to proteins and the nanoprobes. The discrimination ability of this array was tested by analyzing eight proteins at low concentrations. Finally, 100% accuracy was achieved for the identification of 48 unknown protein samples. The summary of quantum dot (QD)-based sensor arrays is shown in Table 5.

## 6. Other Metal Nanoparticle-Based Sensor Arrays

Other metal nanoparticles, such as Fe_3_O_4_ NPs, AgNPs, MoS_2_, and CuS NPs, were also prepared to develop sensor arrays for the discrimination of proteins, bacteria, and cells [78,176,177,178,179,180,181,182,183]. Scientists fabricated dopamine and trimethylammonium functionalized Fe_3_O_4_ NPs, which were able to catalyze the oxidation of colorless ABTS to become a green product in the presence of H_2_O_2_ [66]. When analyte proteins were added into the mixture, the accessibility of reaction substrates to the NP surface was adjusted, leading to a change in the catalytic efficiency. The Fe_3_O_4_ NP-based sensor array can identify ten proteins at a concentration of 50 nM. Cui and co-workers developed a dynamically tunable, low-background, and highly reproducible CL system based on luminol-functionalized silver nanoparticles (luminol-AgNPs) for protein sensing [184]. Qu and Ren also utilized AgNPs to construct sensor arrays for the recognition of proteins [185]. Although AgNPs have some unique properties, their instability and toxicity limit their application in bioanalysis. Ren and Pu developed a sensor array for the identification of proteins and antibiotic-resistant bacteria utilizing CuS NPs and fluorescent dyes [186]. The sensing platform showed excellent discrimination ability between antibiotic-resistant and antibiotic-susceptible bacteria extracts.

Zhang and coworkers constructed quaternized magnetic nanoparticle (q-MNP)-fluorescent polymer systems for the detection and identification of bacteria [187]. The complexes of the q-MNP-fluorescent polymer were disrupted by the bacterial cell membranes, leading to a unique fluorescence response. Eight bacteria were quantitatively discriminated with LDA with an accuracy of 87.5% for 10^7^ cfu/mL within 20 min. The sensor array was also used to identify 32 unknown bacteria samples with an accuracy of 96.8%. The summarization of the other metal nanoparticle-based sensor arrays is shown in Table 6.

## 7. Conclusions

The integration of nanomaterials in optical differential sensors has provided a powerful platform for biosystems analysis [188]. In contrast to traditional lock-and-key biosensing, these sensors function as chemical noses with the ability to recognize a wide range of targets, including proteins, mammalian cells, and microorganisms [189].

The use of nanomaterials has expanded the design possibilities of analysis arrays in several significant ways. Firstly, more different molecular assembly modes and larger assembly quantities are now achievable using covalent bonding modifications or surface adsorption, etc. Secondly, nanomaterials themselves possess more diverse signal outputs, such as the abundant fluorescence signals of quantum dots at various wavelengths or the color changes of nanogold particles. Thirdly, nanomaterials provide a wider range of interaction mechanisms between nanointerfaces and biomolecules, reflecting surface charge and molecular structure, etc. Lastly, the application of hierarchical nanomaterials further enhances the capabilities of analysis arrays. By combining hierarchical nanomaterials, additional advantages for biosensing applications can be achieved. These materials improve signal intensity and enhance various energy transfer processes. The integration of hierarchical nanomaterials alongside other nanomaterials expands the design possibilities of analysis arrays, enabling even more diverse and efficient biosensing platforms [190]. Overall, the application of nanomaterials has dramatically improved the sensitivity and recognition range of pattern recognition detection, leading to more diverse array designs [191]. However, challenges remain in this field. 

Future research directions and urgent issues include: (1) Further theoretical studies are needed to understand the signal mechanisms of most sensing arrays. (2) To radically improve the accuracy of pattern recognition, the stability and controllability of the nanomaterials are critical. (3) Further enhance the discrimination ability and sensitivity of pattern recognition sensors. (4) Efforts should be made to reduce the production cost of nanoprobes to decrease expenses associated with their use. (5) The application of interfacial self-assembly on micro/nanochip technology should be helpful for the high-throughput data collection for next-generation chemical noses. (6) The introduction of novel and superior nanomaterials would greatly improve the performance of the sensor array. For example, single-chirality carbon nanotubes are recently drawing a large amount of research interest for their near-infrared fluorescence signals and specific recognition and binding abilities for biomolecules. Addressing these challenges and capitalizing on emerging advancements will undoubtedly contribute to the continuous progress of this field.

## Figures and Tables

**Table 1:** The main characteristics of the common nanomaterials studied for optical differential sensing.

Nanomaterials	Biological Interaction	Optical Signal
AuNPs	Competitive adsorption, Au-S modification	fluorescence quenching, Colorimetric signal due to aggregation
Graphene oxide (GO)	Competitive adsorption, Modification through -COOH	fluorescence quenching
QDs	Bind nonspecifically via electrostatic interactions	Fluorescence emission with different lengths and high quantum yield

**Table 2:** Development of optical differential sensing based on nanomaterials for biological analysis in different timelines.

Year	Development of Optical Differential Sensing Based on Nanomaterials for Biological Analysis
2007	Rotello’s group developed a sensor array consisting of six non-covalent gold nanoparticle-fluorescent polymer conjugates for identification and quantitative differentiation of proteins [65]
2010	Rotello and co-workers developed enzyme-amplified array sensing (EAAS) with NPs to dramatically increase the sensitivity for protein identification [38]
2012	Rotello and co-workers also achieved colorimetric differentiation of proteins with catalytically active NPs used for both recognition and signal transduction/amplification [66]
2012	Rotello and co-workers also developed gold-nanoparticle green-fluorescent protein (NP−GFP)-based sensor arrays for the identification of mammalian cell types and cancer states [67]
2012	Dravid, Chou, and De developed nanoscale graphene oxide (nGO) as artificial receptors for array-based protein identification [68]
2012	Fan, Hu, and co-workers employed the combination of fluorescently labeled adaptive “ensemble aptamers” (ENSaptamers) and nGOs for high-precision identification of a wide range of bioanalytes, including proteins, cells, and bacteria [69]
2014	Ouyang and co-workers have synthesized novel blue-emitting ColAu NCs and Mac-Au NCs for discriminating proteins [70]
2014	Qu and Ren utilized a sensing array composed of seven luminescent nanodots, combined with graphene oxide, for protein recognition [71]
2015	He and Chang constructed an array-based protein discrimination system by using eight Au NDs as efficient protein receptors and competent signal transducers [72]
2016	Zhang and Tang develop a multicolor quantum dot (QD)-based multichannel sensing platform for rapid identification of multiple proteins [73]
2017	Shi and Wu employed a colorimetric sensor array consisting of four gold nanoparticles (AuNPs) with diverse surface properties for the rapid identification of microorganisms [74]
2018	Pu, Ren and Qu developed a sensitive and effective method for pattern recognition of proteins using nanozyme (g-C_3_N_4_) as a receptor [75]
2022	Li and Han utilized five fluorescent positively charged polymers (P1–P5) and negatively charged graphene oxide (GO) for differentiating between different proteins [76]
2022	Huang, Han and Li utilized three modified polyethyleneimine and negatively charged graphene oxide for differentiating different bacteria [77]
2023	Tian and Wu utilized silver nanoparticles for differentiating proteins in various osmolyte solutions [78]
2024	Yang employed DBCO-UCNPs for the differentiation of different pathogens in terms of phenotyping classification and antibiotic resistance identification [34]

**Table 3:** Summarization of Gold Nanoparticle-Based Sensor Array Construction Strategies with Different Artificial Receptors (ARs).

Nanomaterials	Strategies	Numbers of ARs	Signals	Data Analysis Methods	Analytes	LOD	Ref.
AuNPs	Competitive binding between nanoparticle-polymer complexes and cells	3	Fluorescence	LDA	Cells	n.a.	[90]
AuNPs	Competitive binding between NP-GFP complexes and cells	6	Fluorescence	LDA	Cells	5000 cells	[91]
AuNPs	Proteins displace β-Gal from the β-Gal/AuNP complex to restore its catalytic activity towards the fluorogenic substrate	6	Fluorescence	LDA	Proteins	1 nM	[38]
AuNPs	Competitive binding between GFP and analytes to the particle surface	8	Fluorescence	LDA	Cells, tissues	200 ng/1000 cells	[67]
AuNPs	Different aggregation behaviors and color changes when the aptamer-protected AuNPs mixed with proteins	3	Absorbance	LDA	Proteins	n.a.	[126]
AuNPs	Competitive interactions between bacterial species and the cationic AuNPs,	1	Fluorescence	LDA	Bacteria	n.a.	[149]
Col-Au NCs and Mac-Au NCs	Different interactions between proteins and the Au NCs surface	2	Fluorescence	LDA	Proteins	n.a.	[150]
AuNPs	Differential interactions between DNA-AuNPs and cells result in distinct Au growth reactions	6	Absorbance	LDA	Cells	n.a.	[129]
AuNPs	Competitive binding between DNA and proteins from the surface of AuNPs	3	Fluorescence, Absorbance	LDA, HCA	Proteins	50 nM	[127]
AuNDs	Differential interactions of proteins with AuNDs	8	Fluorescence	LDA, HCA	Proteins	n.a.	[72]
AuNPs	Competitive binding between the fluorescent proteins and the cell lysate analytes to BenzNPs	1	Fluorescence	LDA, HCA	Cells	1000 cells	[92]
AuNPs	Differential interactions of microorganisms and AuNPs caused aggregation of four sensing elements at different degrees	4	Absorbance	LDA	Microorganisms	n.a.	[28]
AuNCs	Differential interactions between free proteins and capping proteins on Au NCs	5	Phosphorescence	LDA, HCA	Proteins	n.a.	[143]
AuNPs	Different proteins triggered the DNA-protected AuNPs to exhibit different aggregation behaviors caused various solution color change	2	Absorbance	LDA, HCA	Proteins	50 nM	[128]
AuNCs	Differential binding between proteins and AuNCs resulting in the fluorescence change of AuNCs	6	Fluorescence	LDA	Proteins, serum	10 nM	[144]
AuNCs	Differential interactions between the protein and the metal ion-AuNCs	6	Fluorescence	LDA	Proteins, bacteria	n.a.	[146]
AuNPs	Aggregation of AuNPs induced by the differential metabolic capabilities of bacteria towards D-amino acids (D-AAs)	3	Absorbance	LDA, HCA	Bacteria	n.a.	[130]
AuNCs	Different oxidation of AuNCs@His by ROS	3	Fluorescence	PCA, HCA	Cells	n.a.	[147]
AuNCs	Fluorescence intensity of AuNCs was quenched to varying degrees by the bacteria	3	Fluorescence	LDA, HCA	Bacteria	10^5^ CFU/mL	[148]

**Table 4:** Summarization of Graphene Oxide (GO)-Based Sensor Arrays Construction Strategies with Different Artificial Receptors (ARs).

Nanomaterials	Strategies	Numbers of ARs	Signals	Data Analysis Methods	Analytes	LOD	Ref.
nGO	Proteins displace fluorophores from the nGO surface through binding competition	5	Fluorescence	LDA	Proteins	10 nM	[68]
nGO	Competitive binding between ssDNA-nGO complexes and analytes	7	Fluorescence	LDA	Proteins, cells and bacteria	5 μM	[69]
GQDs, QDs, CDs-COOH, PEI-CDs, BSA-AuNCs, Lys-AuNCs, AgNCs and GO	Competitive binding between luminescent nanodots and analytes to GO surfaces	7	Fluorescence	LDA	Proteins, bacteria	n.a.	[71]
GQDs-COOH, GQDs-NH_2_, PEI-CDs, QDs, BSA-AuNCs, Lys-AuNCs and GO	Competitive binding between luminescent nanodots and cells to GO surfaces	6	Fluorescence	LDA	Cells	200 cells	[153]
GO	Competitive interaction among GO, AIEgen and biomolecules	7	Fluorescence	PCA	Microbes	n.a.	[167]
GO	Competitive interaction among GO, fluorescent polymers and proteins	5	Fluorescence	LDA	Proteins	n.a.	[76]
GO	Competitive binding between bacteria and GO with fluorescent PEIs	1	Fluorescence	LDA	Bacteria	OD_600_ = 0.125	[77]

**Table 5:** Summarization of Quantum dot (QD)-based Sensor Arrays Construction Strategies with Different Artificial Receptors (ARs).

Nanomaterials	Strategies	Numbers of ARs	Signals	Data Analysis Methods	Analytes	LOD	Ref.
Mn–ZnS QDs	Different interactions of Mn–ZnS QDs with proteins	1	Fluorescence phosphorescence light scattering	PCA	Proteins	0.5 µM	[173]
QDs	Differential competitive and selective non-covalent interactions between nanoparticles and cell surface	2	Fluorescence	LDA	Cells	10,000 cells	[100]
CdTe QDs	Differential interactions between analytes and IL@CdTe QDs	5	Fluorescence	LDA	Proteins, bacteria	10 nM	[175]
CuO NPs, ZnO NPs, Eu_2_O_3_ NPs, AuNPs, AgNPs, Au-Ag core-shell and CdSe QDs	Protein presence disrupts nanoparticle-QD interactions, resulting in fluorescence turn on or further quenching	6	Fluorescence	LDA	Proteins, cells	5 µM	[174]

**Table 6:** Summarization of the Sensor Arrays Construction Strategies with Different Artificial Receptors (ARs).

Nanomaterials	Strategies	Numbers of ARs	Signals	Data Analysis Methods	Analytes	LOD	Ref.
Fe_3_O_4_ NPs	Differential interactions of proteins with Fe_3_O_4_ NPs affected the accessibility of ABTS to the NP surface	2	Fluorescence	LDA	Proteins	50 nM	[66]
AgNPs	Different adsorption capacity of proteins onto luminol-AgNPs affected the accessibility of H_2_O_2_ to the NPs surface	1	Chemiluminescence	PCA	Proteins	n.a.	[184]
Quaternized magnetic nanoparticles (q-MNP)	Competitive binding between fluorescent polymer and bacteria to GO surfaces q-MNP	3	Fluorescence	LDA	Bacteria	n.a.	[187]
CuS NPs	Competitive binding between analytes and fluorescent dyes towards CuS NPs	4	Fluorescence	PCA	Proteins, bacteria	n.a.	[186]
AgNPs	The diversity in structure and properties of various proteins results in different effects on the synthesis of AgNPs under light irradiation, leading to AgNPs with distinct LSPR absorption spectra	3	Absorbance	PCA	Proteins	n.a.	[185]
DBCO-UCNPs	Different bacteria exhibit differences in metabolic capability, sensitivity to antibiotics, and surface properties and thus lead to discriminative responses	6	Fluorescence	PCA, HCA, LDA	Bacteria	10^5^ CFU/mL	[34]

## Data Availability

Not applicable.
